# Supplementary material for: FERN – a Java framework for stochastic simulation and evaluation of reaction networks
Source: BMC Bioinformatics. 2008 Aug 29;9:356. doi: 10.1186/1471-2105-9-356 (PMC2553347; doi:10.1186/1471-2105-9-356)
Supplement: Additional file 1 — FERN distribution, Version 1.3. This archive contains the FERN source code and binaries as well as documentation and example models in FernML and SBML. [file 1471-2105-9-356-S1.zip › fern/doc/javadoc/fern/network/modification/package-summary.html]

fern.network.modification


---


|  |  |  |
| --- | --- | --- |
| **Overview** \| **Package** \| Class \| **Use** \| **Tree** \| **Deprecated** \| **Index** \| **Help** | |  |
| **PREV PACKAGE**   **NEXT PACKAGE** | **FRAMES**    **NO FRAMES**     **All Classes** |  |


---

## Package fern.network.modification

Provides classes for modifications of networks.

**See:**
  
          **Description**

| **Class Summary** | |
| --- | --- |
| **CatalysedNetwork** | Modifies the network by adding reactions X+C -> Y+C (where C is each catalyst of the original reaction). |
| **ExtractSubNetwork** | Extracts some reactions / species from a given net to form a new network. |
| **ModifierNetwork** | Base class for modified networks, which implements the full `Network` interface. |
| **ReversibleNetwork** | Doubles each reaction in a way that each original unidirectional reaction becomes reversible.As proposed by `ModifierNetwork`, the reactions are not copied but the indices are redirected. |

## Package fern.network.modification Description

Provides classes for modifications of networks.

---


|  |  |  |
| --- | --- | --- |
| **Overview** \| **Package** \| Class \| **Use** \| **Tree** \| **Deprecated** \| **Index** \| **Help** | |  |
| **PREV PACKAGE**   **NEXT PACKAGE** | **FRAMES**    **NO FRAMES**     **All Classes** |  |


---
